# The Many Faces of IpaB

**DOI:** 10.3389/fcimb.2016.00012

**Published:** 2016-02-09

**Authors:** Wendy L. Picking, William D. Picking

**Affiliations:** Department of Pharmaceutical Chemistry, School of Pharmacy, University of KansasLawrence, KS, USA

**Keywords:** *Shigella*, type III secretion, IpaB, invasion plasmid antigen, pathogenesis

## Abstract

The type III secretion system (T3SS) is *Shigella*'s most important virulence factor. The T3SS apparatus (T3SA) is comprised of an envelope-spanning basal body and an external needle topped by a tip complex protein called IpaD. This nanomachine is used to deliver effector proteins into host cells to promote pathogen entry. A key component of the matured T3SS needle tip complex is the translocator protein IpaB. IpaB can exist in multiple states when prepared as a recombinant protein, however, it has also been described as having additional roles in *Shigella* pathogenesis. This mini-review will briefly describe some of the features of IpaB as a T3SS needle tip protein, as a pore-forming translocator protein and as an effector protein. Reflection on the potential importance of the different *in vitro* states of IpaB on its function and importance in serotype-independent vaccines is also provided.

## Introduction

### Shigella

*Shigella* species (*S. flexneri, S. dysenteriae, S. sonnei*, and *S. boydii*) are etiologic agents of shigellosis, a potentially life-threatening bacillary dysentery. While shigellosis is recognized as a disease of the developing world (Sansonetti, 2006; Kotloff et al., 2013), it is also an underreported problem in industrialized regions where outbreaks are not uncommon (Sjölund Karlsson et al., 2013; Thompson et al., 2015). Following ingestion of contaminated water, *Shigella* travels to the colon where it invades the intestinal epithelium (Adam and Picking, 2015). Infection is initiated when *Shigella* crosses M cells and invades/kills resident macrophages in the associated lymphoid tissues (Zychlinsky et al., 1992). This provides the bacterium with access to the basal side of the colonic epithelium. From here *Shigella* invades the overlying epithelial cells by macropinocytosis followed by vacuolar escape into the host cytoplasm where it replicates and directly invades neighboring cells (Carayol and Tran Van Nhieu, 2013; Tran Van Nhieu and Sansonetti, 2015). *Shigella*'s ability to kill macrophages and invade epithelial cells is key to its virulence.

### The gram negative bacterial type III secretion system (T3SS)

Many Gram negative pathogens possess type III secretion systems (T3SS; Burkinshaw and Strynadka, 2014; Portaliou et al., 2015). The T3SS allows communication with eukaryotic cells to subvert normal cellular processes for the pathogen's benefit. The T3SS apparatus (T3SA) is a complex nanomachine used to deliver bacterial effector proteins directly into and across eukaryotic cell membranes. The architecture of the T3SA is well conserved among otherwise diverse bacteria. In contrast, the effector proteins that are responsible for subverting host cell activities are pathogen specific with regard to their functions and roles in pathogenesis (Bulgin et al., 2010; Plano and Schesser, 2013; Raymond et al., 2013). Three of the best characterized T3SA structures belong to *Shigella, Salmonella* and *Yersinia* (Kubori et al., 1998; Mueller et al., 2005; Kawamoto et al., 2013; Hu et al., 2015). Among these, the T3SA of *Shigella* and that encoded by the *Salmonella* Pathogenicity Island 1 (SPI-1) are the most closely related with respect to overall structure and the structure of the controlling needle tip complex that is described below.

### A brief overview of the *Shigella* T3SA structure

**Figure 1:**
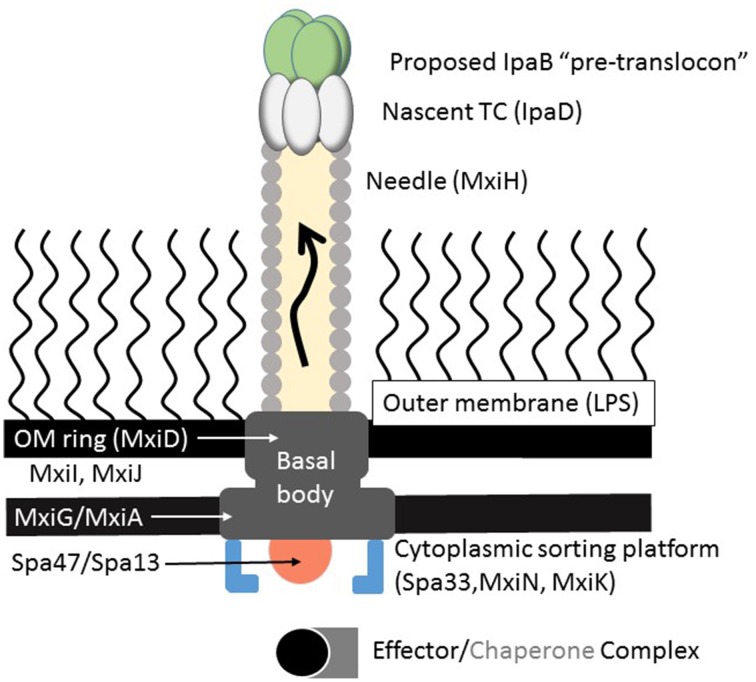
**A schematic of the type III secretion apparatus (T3SA) is shown**. For *Shigella*, the cytoplasmic sorting platform contains multiple copies of Spa33, MxiN, and possibly MxiK. The platform is proposed to house the Spa47 ATPase and the Spa13 stalk that links it to the major export protein MxiA. Within the inner membrane are MxiJ and MxiG, the latter of which may promote interaction with the proteins of the sorting platform (e.g., Spa33). Located within the outer membrane and spanning the periplasm, respectively, are the OM ring protein MxiD and the inner rod protein MxiI. External to the outer membrane and spanning the O antigen layer of the LPS is the needle which is composed of many copies of MxiH which are assembled in a helical manner to provide a conduit from the basal body to the extracellular milieu. Capping the needle and controlling type III secretion in response to extracellular signals is the tip complex (TC). It is proposed that the TC of newly formed needles is comprised of five copies of IpaD, however, a TC containing four copies of IpaD and one of IpaB has been suggested. Following maturation (as caused by exposure to bile salts, for example), IpaB is recruited to the T3SA needle tip and it is from this position that it contributes to control of type III secretion by sensing contact with a host cell either through the recognition of receptor proteins or insertion into the host cell membrane. In the absence of IpaC, IpaB is still capable of inserting into target cell membranes to form what may be described as the pre-translocon pore. In the presence of IpaC, IpaB penetration of the host membrane promotes full secretion induction and formation of the translocon pore through which effector proteins pass.

Much of what is known of the T3SA structure stems from transmission electron microscopy studies of *S. flexneri* and *Salmonella* Typhimurium (Kubori et al., 1998; Blocker et al., 1999; Tamano et al., 2000; Sani et al., 2007; Veenendaal et al., 2007; Lara-Tejero et al., 2011). The overall T3SA architecture resembles a syringe and needle that creates a conduit from the bacterium to the host cell interior (Figure 1). The T3SA is composed of over 25 different protein with many dedicated to forming the basal body which spans the bacterial envelope. This structure possesses a cytoplasmic bulb or sorting platform (see Figure 1) that controls the hierarchy of secretion needed for apparatus assembly and the controlled release of effectors (Lara-Tejero et al., 2011; Hu et al., 2015). In *Shigella*, the ATPase Spa47 (similar to the β subunit of the F_0_F_1_ ATPases) provides a portion of the catalytic power of the T3SS (Johnson and Blocker, 2008) and is involved in separating effector proteins from their cognate chaperones within the soring platform prior to effector secretion (Lara-Tejero et al., 2011). The remaining structural components of the injectisome basal body consist of: (a) rings imbedded in the cytoplasmic membrane; (b) inner rod proteins that span the periplasm; and (c) outer membrane ring proteins that connect the base with the extracellular portions of the T3SA (Figure 1). There are reviews available that describe the detailed structural and functional roles of the basal body components (e.g., Burkinshaw and Strynadka, 2014; Portaliou et al., 2015).

Extending outward from the T3SA base is a needle that crosses the outer membrane with an exposed tip complex (TC; Kenjale et al., 2005; Picking et al., 2005; Deane et al., 2006; Espina et al., 2006; Olive et al., 2007; Epler et al., 2012; Fujii et al., 2012). The TC is exposed to the extracellular environment where it has a role in sensing the signals that lead to secretion induction (i.e., host cell contact). The *Shigella* needle is ~45 to 50 nm in length, with an outer diameter of 7 nm and a 2–3 nm wide inner channel (Deane et al., 2006; Shen et al., 2012). Based on low-resolution electron microscopy images of the *Shigella* needle, the monomers that make up the needle pack in a helical fashion to give rise to an extended cylinder with an architecture of 5.6 subunits per turn and a 24Å helical pitch (Cordes et al., 2003). The TC that caps the T3SA needle consists of five copies of the protein IpaD (Picking et al., 2005; Espina et al., 2006; Epler et al., 2012), which is consistent with a report that the *Yersinia* TC contains five copies of the IpaD homolog LcrV (Mueller et al., 2005). Under the appropriate conditions, the TC also contains IpaB (Olive et al., 2007; Veenendaal et al., 2007; Cheung et al., 2015), which is a hydrophobic protein that assumes different structural states that relate to its multiple roles in *Shigella* virulence (see below).

### The role of the needle tip complex in regulating secretion

Three *Shigella* T3SA proteins that contribute to secretion control from an extracellular location are MxiH (needle monomer; Kenjale et al., 2005), IpaD (the nascent needle TC; Picking et al., 2005; Olive et al., 2007; Stensrud et al., 2008; Roehrich et al., 2013) and IpaB (part of the mature TC following exposure to bile salts; Olive et al., 2007; Epler et al., 2009; Roehrich et al., 2010). A *mxiH* null mutation eliminates type III secretion due to loss of the needle, however, an *ipaD* or *ipaB* null mutation results in uncontrolled hypersecretion (Ménard et al., 1993, 1994a). This suggests a central role for each in sensing the extracellular signals responsible for secretion induction. The functional roles of IpaD and IpaB in sensing the environment, in combination with their localization at the *Shigella* surface, suggests they can be used as antigens to induce a protective/neutralizing immune response (Martinez-Becerra et al., 2012, 2013a). This is consistent with the observation that antibodies against the *Yersinia* TC protein LcrV (Mueller et al., 2005) neutralize its function of delivering Yop proteins (Cowan et al., 2005). In fact, IpaD and IpaB are now both being successfully explored as targets for generating a serotype-independent subunit vaccine to prevent shigellosis (Martinez-Becerra et al., 2012, 2013b).

As mentioned, *Shigella* IpaD has been shown to be important for controlling type III secretion and this appears to also be true for its *Salmonella* homolog SipD (Ménard et al., 1993; Kaniga et al., 1995; Picking et al., 2005; Veenendaal et al., 2007; Roehrich et al., 2013). This may also be the case for LcrV from *Yersinia* since it provides the scaffold for Yop translocon formation (Broz et al., 2007). Because IpaB is part of the TC under certain conditions, it is probably from this position that it contributes to secretion control (Olive et al., 2007; Veenendaal et al., 2007; Shen et al., 2010; Cheung et al., 2015). Since IpaB is known to be a cholesterol binding protein (Lafont et al., 2002; Hayward et al., 2005; Epler et al., 2009) and binds proteins associated with lipid rafts such as integrins and CD44 (Watarai et al., 1996; Skoudy et al., 2000), there is consensus that IpaB recognizes host cell contact as part of the T3SA needle TC. IpaB is thus important in controlling type III secretion as part of the T3SA needle TC (Epler et al., 2009) and it is possible that its C terminus is directly involved in this process (Roehrich et al., 2010).

### Cytoplasmic IpaB forms a heterodimer with IpgC

IpaB contains notable hydrophobicity which renders it unstable when expressed in the absence of its chaperone IpgC (Menard et al., 1994b; Birket et al., 2007). IpgC binds to a defined region near the IpaB N terminus (between residues 11 and 76) and has been proposed to bind a region near the IpaB C terminus (Page et al., 2001; Birket et al., 2007; Lunelli et al., 2009; Lokareddy et al., 2010; Adam et al., 2012). IpgC binding leads to the formation of a heterodimer in the bacterial cytoplasm (Birket et al., 2007; Adam et al., 2012), though a heterotrimer containing two IpgC and one IpaB has been proposed (Lokareddy et al., 2010). Chaperone association stabilizes IpaB and renders it soluble in aqueous solution (Birket et al., 2007). Removal of IpgC probably occurs as the IpaB/IpgC complex docks at the cytoplasmic sorting platform of the *Shigella* T3SA (Lara-Tejero et al., 2011; Hu et al., 2015). Released IpgC is retained in the bacterial cytoplasm where it forms IpgC homodimers that associate with MxiE to become an active transcription factor to promote late T3SS effector expression (Mavris et al., 2002; Pilonieta and Munson, 2008; Bongrand et al., 2012). Removal of the chaperone for recombinant IpaB and IpgC co-expressed in *E. coli* can be accomplished using mild detergents (Dickenson et al., 2013b). Interestingly, different detergents influence the released IpaB in different ways. The effect of detergents on the secondary, tertiary and quaternary structures (and the membrane active behavior) of IpaB is discussed below.

### IpaB the translocator

Beyond the needle and TC of the *Shigella* T3SA lies the translocon that forms in the host cell membrane following bacterial contact (Blocker et al., 1999; Veenendaal et al., 2007). Translocon formation occurs concomitant with full induction of type III secretion (Lafont et al., 2002; Epler et al., 2009). Bile salts promote recruitment of IpaB into the T3SA needle TC, apparently as a result of direct binding of bile salts by IpaD at the needle tip (Olive et al., 2007; Stensrud et al., 2008). The defining point at which the TC ends and the translocon begins is not entirely clear. Some groups have observed the translocon proteins IpaB and IpaC as part of the mature TC prior to host cell contact (Veenendaal et al., 2007), however, the conditions to which the bacteria are exposed may have a role in dictating the observed TC composition (Olive et al., 2007; Stensrud et al., 2008; Epler et al., 2009; Cheung et al., 2015). A number of studies on *Shigella* have not found IpaB or IpaC as part of the TC (Espina et al., 2006; Sani et al., 2007) and thus far no translocator protein has been found to be part of any other T3SA needle TC complex (e.g., in *Yersinia*; Mueller et al., 2005).

Once formed, the translocon pore is comprised of IpaB and IpaC. Its properties have been analyzed following insertion into erythrocytes using contact-mediated hemolysis (Blocker et al., 1999; Picking et al., 2005; Veenendaal et al., 2007). In the absence of IpaD or IpaB, translocons do not form and *Shigella* is rendered completely unable to lyse erythrocytes (Blocker et al., 1999; Picking et al., 2005). In the absence of IpaC, however, *Shigella* remains ~10% hemolytic, suggesting that IpaB is inserted into erythrocyte membranes in IpaC's absence where it is still able to form a pore and/or cause membrane damage (Blocker et al., 1999; Adam et al., 2014).

### IpaB's *in vitro* pore-forming ability

As part of the translocon, IpaB and IpaC both contribute to pore formation, however, IpaB is also capable of forming pores when prepared as a purified recombinant protein (Senerovic et al., 2012; Dickenson et al., 2013b). Recombinant IpaB co-expressed with its chaperone IpgC is readily separated from its chaperone using detergents (Menard et al., 1994b; Hume et al., 2003; Birket et al., 2007; Senerovic et al., 2012; Dickenson et al., 2013b), however, the detergent used has a significant impact on IpaB's behavior in solution. When prepared in n-octyl-polyoxyethylene (OPOE), IpaB forms stable tetramers, but when prepared in lauryldimethylamine oxide (LDAO) it forms monomers (Dickenson et al., 2013b). While OPOE has a range of molecular sizes due to its chemical heterogeneity (Dickenson et al., 2013b), LDAO is a single Zwitterionic molecule [H_25_C_12_-N(CH_3_)_2_O] that has a critical micelle concentration (CMC) about tenfold lower than OPOE. Perhaps related to their different CMCs, IpaB can be maintained at a high concentration in 0.05% LDAO as opposed to 0.5% OPOE (Dickenson et al., 2013b). Reducing either detergent to below their relative CMCs results in IpaB aggregation.

Both tetrameric and monomeric IpaB retain a strongly α-helical secondary structure (Dickenson et al., 2013b), however, it appears that IpaB prepared in LDAO assumes a structure that is somewhat characteristic of a molten globular state (Chen et al., 2015). Interestingly, while IpaB prepared in either of these detergents associates with phospholipid membranes, only tetrameric IpaB causes the size-dependent release of fluorescent molecules trapped in liposomes (Dickenson et al., 2013b). This suggests that IpaB prepared in OPOE has pore-like properties following insertion into phospholipid membranes.

IpaB has also been described as forming alternative oligomeric forms depending upon the detergent composition of the solution (Hume et al., 2003; Senerovic et al., 2012). When prepared in OPOE which is subsequently removed, IpaB forms trimers that efficiently bind cholesterol and insert into cultured cells in a cholesterol-dependent manner (Hume et al., 2003). This occurs without disruption of the cell membrane, however, this complex also promotes the fusion of liposomes when tested *in vitro*. When IpaB is prepared in LDAO and then the detergent concentration reduced to below its CMC, it oligomerizes in some cases with the resulting oligomers being considerably larger than the tetramers observed in OPOE (Senerovic et al., 2012) or the timers seen after removal of OPOE (Hume et al., 2003). In this case, the oligomers are decamers that can form cation channels in cultured macrophages that are triggered to undergo caspase-mediated pyroptosis (Senerovic et al., 2012). How this relates to possible IpaB effector functions is addressed in the next section.

Unlike the above decameric complexes, IpaB tetramers formed in OPOE are more like what might be expected in translocon pore formation since the approximate cutoff of molecules released from liposomes in their presence is in the range of 3–5 nm, which is similar to the 2.5 nm translocon pore diameter previously described (Blocker et al., 1999). Following crystal structure determination for a large segment of the IpaB N terminus (Barta et al., 2012) and determination that this region associates with IpaD (Dickenson et al., 2013a), it is possible that the tetramer represents IpaB as it exists within the T3SA needle TC. The N-terminal region forms a coiled-coil that may anchor the tetramer to IpaD at the needle tip which would allow insertion of IpaB into the host cell membrane via its exposed hydrophobic domain (Barta et al., 2012; Adam et al., 2014). This would form the progenitor of the translocon pore (pre-translocon pore) and be consistent with the structural similarity seen between the IpaB (and *Salmonella* SipB) N-terminal coiled-coil and the tether portion of bacterial colicins (Barta et al., 2012). It would also be consistent with the cholesterol-binding (Lafont et al., 2002; Hayward et al., 2005) pore-forming toxins (Senerovic et al., 2012) to which IpaB has been compared (High et al., 1992).

### IpaB effector function

IpaB is typically referred to as a *Shigella* T3SS translocator protein, however, it has additional roles in *Shigella* pathogenesis. IpaB has been described as an integral component of the T3SA needle TC, whether as a stable component or a transient component during transition from quiescent to mature T3SA. As a TC component, IpaB is likely to recognize the host cell surface, either through lipid raft-associated protein receptors or through lipids (cholesterol or sphingolipids) to promote its own insertion via an internal hydrophobic domain. An additional important function identified early in the study of IpaB was its ability to induce apoptosis (revised later to pyroptosis) in macrophages due to activation of caspase 1 (Zychlinsky et al., 1992, 1994). This occurs through a direct interaction of IpaB with caspase 1 (Hilbi et al., 1998; Guichon et al., 2001) which results in the release of IL-1β as a precursor to the inflammation responsible for the pathology of shigellosis (Guichon et al., 2001). As noted above, in parallel with caspase activation IpaB may form cation channels that further lead to macrophage pyroptosis via caspase-mediated release of IL-1β (Senerovic et al., 2012). Its ability to influence cholesterol relocation within host cells has also been shown to adversely affect Golgi function (Mounier et al., 2012). Thus, in addition to its roles in sequestering the transcription activator IpgC prior to secretion from the *Shigella* cytoplasm, contributing to the structure of the mature T3SA needle TC, and forming the translocon pore, IpaB also has a role in promoting macrophage killing as a T3SS effector. The macrophage killing then promotes the release of *Shigella* from macrophages beneath the colonic epithelium to allow *Shigella* invasion of colonic epithelial cells.

## Concluding remarks

**Figure 2:**
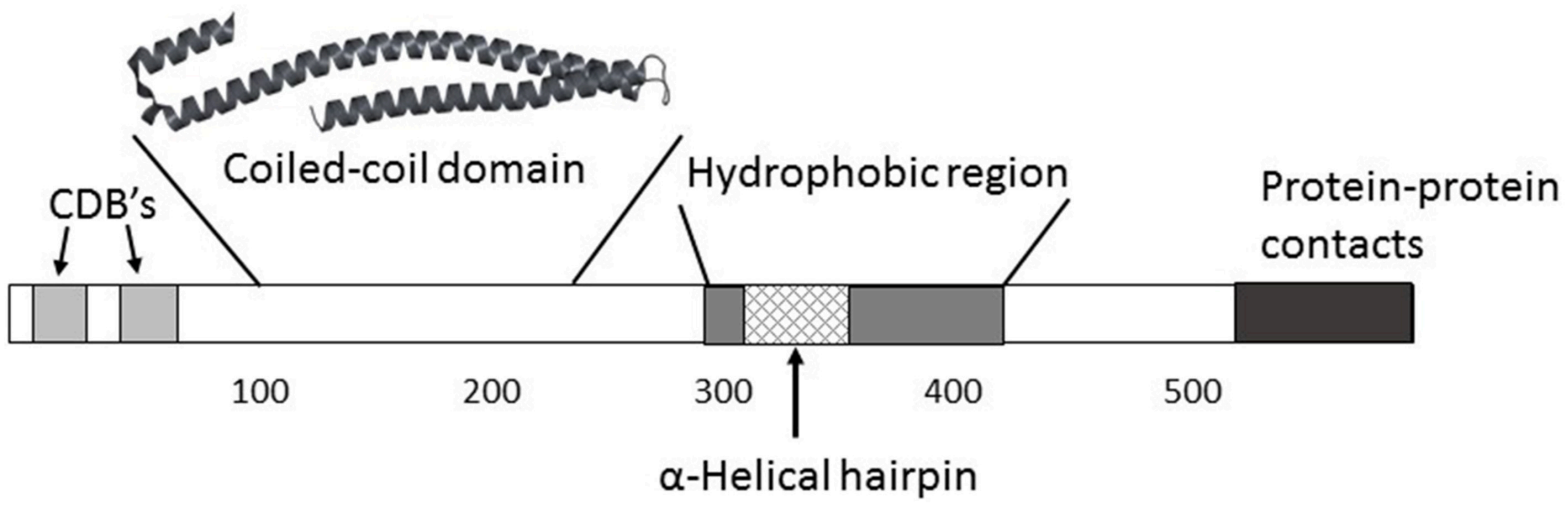
**While a high-resolution structure of full-length IpaB (580 amino acids) is not yet available, an understanding of its functional organization is taking shape**. Two major chaperone-binding domains (CBD's) have been located between residues 11 and 76 near the IpaB N terminus. Additional regions that may be involved in mediating IpaB interactions with itself and possibly other proteins are located near the C terminus. The one region for which there is a high resolution structure lies between residues 120 and 224 which forms a highly elongated coiled-coil. A remarkably similar structure spans residues 80–226 in SipB, the *Salmonella* homolog of IpaB, and similar structures are predicted within the homologs EspD (Enteropathogenic *E. coli*) and YopB (*Yersinia* spp.). This coiled-coil may have a role in anchoring IpaB to IpaD at the T3SA needle tip which would allow IpaB to position its hydrophobic domain for contact with a host cell, not unlike what occurs for membrane-interacting colicins. A large hydrophobic domain lies mostly within the C-terminal half of IpaB and it contains a predicted α-helical hairpin similar to what has been observed in some membrane-interacting colicins. The region involved in IpaB's ability to associate with and activate caspase 1 is reported to also lie within this region (between residue 316 and 401).

IpaB is clearly a remarkable protein having multiple roles during the course of *Shigella* infection. Figure 2 outlines some of what is known of the functional organization of IpaB. Some of these roles may be directly related to the different structural states IpaB can assume *in vitro*. Further high-resolution structural information will be needed for purified recombinant IpaB and *in situ* (TC-localized) IpaB before this will be known with certainty. Nevertheless, because of its central role as a virulence factor coupled with its surface exposure (along with IpaD) at the surface of *Shigella*, IpaB is a potential target for prevention of shigellosis. Thus, while the multifunctional capacity of IpaB provides an excellent target for understanding *Shigella* virulence, it is also being used for developing strategies to prevent shigellosis.

## Author contributions

The drafting of this work was by WDP. Final approval of the manuscript to be published was by WDP and WLP. WDP and WLP agree to be accountable for all aspects of this work.

## Funding

Studies in the Picking laboratories have been funded by the National Institutes of Health (R01 AI099489) and the University of Kansas.

### Conflict of interest statement

The authors declare that the research was conducted in the absence of any commercial or financial relationships that could be construed as a potential conflict of interest.
